# Influence of Vitamin D and Its Analogues in Type-B Lymphomas

**DOI:** 10.3390/curroncol32030135

**Published:** 2025-02-26

**Authors:** Valerio Basile, Alessandro Allegra, Herbert Ryan Marini, Massimiliano Berretta, Barbara Granata, José Freni, Domenico Puzzolo, Fabio Stagno, Paola Midiri, Valentina Urzì Brancati, Letteria Minutoli

**Affiliations:** 1 Department of Clinical and Experimental Medicine, University of Messina, 98125 Messina, Italy; valeriobasile87@hotmail.com (V.B.); massimiliano.berretta@unime.it (M.B.); barbaragranata@live.it (B.G.); mdrpla94d70f158n@studenti.unime.it (P.M.); valeurzi@hotmail.it (V.U.B.); lminutoli@unime.it (L.M.); 2 Division of Hematology, Department of Human Pathology in Adulthood and Childhood, University of Messina, 98125 Messina, Italy; alessandro.allegra@unime.it (A.A.); stagnof@unime.it (F.S.); 3 Department of Biomedical and Dental Sciences and Morphofunctional Imaging, University of Messina, 98125 Messina, Italy; jose.freni@unime.it (J.F.); puzzolo@unime.it (D.P.)

**Keywords:** type B-lymphoma, vitamin D, vitamin D analogs, scoping review

## Abstract

Lymphomas represent a heterogeneous group of blood tumors, generally divided into non-Hodgkin lymphoma (NHL) (90% of all lymphomas) and Hodgkin lymphoma (HL). High-grade NHL can rapidly progress so that new strategies and potentially therapeutical options are needed. Recently, it was shown that Vitamin D (VitD) inhibits the growth of cancer cells, controls their invasion and metastasis, and strengthens the antitumor activity of various types of chemotherapeutic anticancer agents. Therefore, we reviewed the recent literature about the influence of VitD and its analogues (VDAs) on the treatment and the prognosis of B-cell lymphomas. As to the in vitro studies in different cell lines, VitD3 and VDAs enhanced the anti-proliferative efficacy of various chemotherapeutics and increased the expression of VitD receptor. In in vivo studies, blood levels of VitD were considered: higher values of plasma bioavailable VitD were correlated with better progression-free survival (PFS) and overall survival (OS), while an unfavorable PFS and OS were observed in VitD deficient groups. No clinical trial was made on the analogs, thus confirming the absence of in vivo positive role of these synthetic drugs. In conclusion, higher levels of circulating VitD are related to improved OS, reduced cancer-specific mortality, and better disease-free survival. VitD and analogs showed also positive effects in in vitro studies, while only VitD was able to improve clinical parameters. Furthermore, a complex approach with plant-based diet, adequate levels for motor exercise, and/or eventual VitD supplementation could be a valuable strategy to challenge lymphomas.

## 1. Introduction

Lymphomas, together with leukemias and myelomas, represent a heterogeneous group of blood tumors, originating from hematopoietic stem cell mutations or alteration in progenitor cells already oriented in a lymphoid direction. Lymphomas are generally divided into non-Hodgkin lymphoma (NHL) (90% of all lymphomas) and Hodgkin lymphoma (HL). NHL is further divided into B-cell, T-cell, and natural killer (NK) cell types, while HL is divided into classical and non-classical types [1,2,3]. Moreover, according to a clinical approach, lymphomas are of high grade (aggressive) or low grade (indolent). From an epidemiological point of view, indolent lymphomas are much more frequent than aggressive ones [4,5]. Furthermore, the latest classification of the World Health Organization (WHO) identified more than 80 lymphoma types and grouped them into three main classes based on their morphology, immunophenotype, genetic and molecular lesions profiles, clinical characteristics, and cellular derivation [3,4]. About 95% of the lymphomas are of B-cell origin, being the other T/NK-cell malignancies.

The Global Cancer Statistics 2022 from the International Agency for Research on Cancer (IARC) reported 20 million new cancer cases in 2022; amongst these, 553.010 were new cases of NHL and 82,409 new cases of HL, each with a mortality rank of 250.475 and 22.701 [6].

In last decades, an increase in the incidence of some subtypes of type B lymphomas, such as diffuse large B-cell lymphoma (DLBCL), has been observed [6,7]. This could be due to a combination of factors, including better recognition and diagnosis of lymphomas, as well as changes in environment and lifestyle. Several non-modifiable or modifiable risk factors are involved in the pathogenesis of B-cell lymphomas, such as tobacco and alcohol consumption, obesity, family history, autoimmune diseases, infections (including Epstein-Barr virus, Helicobacter pylori, hepatitis C virus), and inflammatory diseases [5,7]. However, other molecular factors also contribute to the pathogenesis of these types of tumors, such as the expression of B-cell receptor, crucial for the survival or proliferation of most malignant B-cells, the interaction of these transformed cells with other cells in the tumor microenvironment, and the recognition of an antigen that contributes to the survival and proliferation of B lymphoma cells [8].

NHLs have a wide range of histological aspects and clinical features, and patients commonly present lymphadenopathy or splenomegaly. In general, these heterogeneous features could make difficult the diagnosis, and timely identification is important because different therapies are available. In fact, the treatment of B-cell lymphomas depends on the specific subtype and staging of the disease [5]. High-grade NHL can rapidly progress, and prompt treatment is required, usually with a combination of chemotherapy with rituximab, a monoclonal antibody against CD20 B-cell specific surface antigen. Indolent lymphomas are not considered curable with conventional therapy. However, patients presenting localized lymphadenopathy may be treated by radiotherapy [5].

Thanks to advances in cancer therapy, many patients with different types of B lymphomas respond well to therapies and have good long-term survival prospects [9]. However, at present, lymphoid cancer treatments are not very successful. Therefore, new strategies and potentially therapeutical options are needed to increase efficiency and tolerance, compared to standard treatments, such as chemotherapy, immunochemotherapy with an antibody–drug conjugate or bispecific antibodies, or gene and cell therapies [10,11,12].

Recently, preclinical and epidemiological data have suggested that the main circulation form of Vitamin D (VitD), 25(OH)2D_3_, plays a crucial role in the pathogenesis, progression, and therapy of hematological cancers, and it was shown that patients with hematological diseases have lower serum levels of VitD3 [13]. In general, people with a lower VitD3 serum level have shown a higher risk of developing different types of cancer [11,14,15]. Although VitD3 primary function is the regulation of calcium and phosphate metabolism, its compounds inhibit the growth of cancer cells and control their invasion and metastasis through the induction of cell-cycle arrest, the control of apoptosis, the differentiation of cells, the reduction in angiogenesis, and the modulation of pro-inflammatory cytokine production [14,16]. Interestingly, in vitro and in vivo preclinical analyses indicated that VitD3 strengthens the antitumor activity of various types of chemotherapeutic anticancer agents, such as platinum analogues [17,18] and taxanes [18]. Moreover, it also increases the antitumor effect of cytotoxic biologic agents, such as dovitinib, a multi-kinase inhibitor [19].

However, although the significant anticancer properties displayed by VitD, unfortunately the high doses of administration necessary for its adjuvant anticancer effect could lead to hypercalcemia, an unfavorable condition for patients. In this regard, numerous clinical trials have demonstrated a better tolerability and promising results of several synthetic VitD analogues (VDAs), supporting a further investigation of their clinical utility, especially in combination with standard treatments, such as chemotherapeutic drugs and immunotherapy [20,21]. Therefore, the purpose of our study was to review the recent literature about the influence of VitD and its analogues on the treatment and the prognosis of B-cell lymphomas.

## 2. Materials and Methods

**Figure 1:**
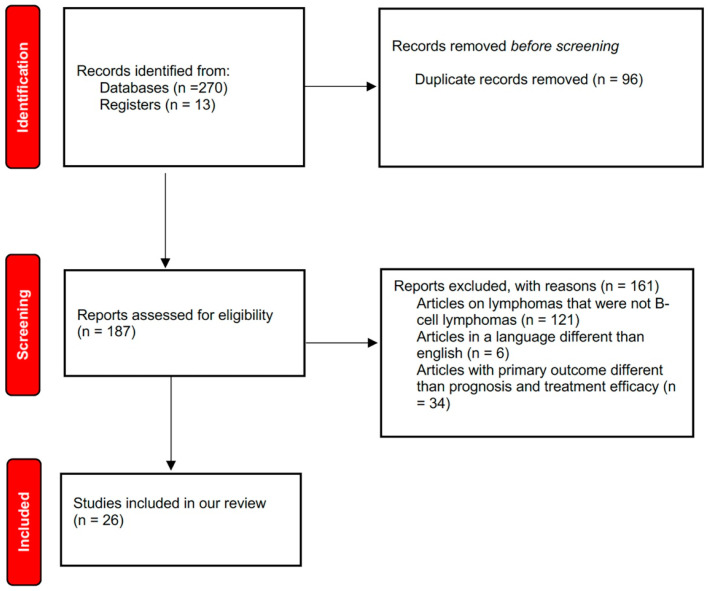
Flow diagram of literature search.

We searched PubMed and Medline databases to find all relevant English-language scientific papers dealing with the effects of VitD and its analogs on B lymphomas. Inclusion criteria were full texts entirely in English language with an abstract and at least one of the following features: clinical and/or preclinical studies on the role of VitD or VDAs in patients with B lymphomas; in vitro studies on the role of VitD or VDAs in B lymphoma cells. The latest studies, both in vitro and in vivo, were selected. We excluded studies written in a language different from English and case reports. Boolean operators AND/OR were used to combine search terms. The following search strings were used: “Vitamin D” OR “Vit D analogues” AND “lymphomas” OR “25-hydroxyvitamin D” AND “lymphomas”. V.U.B., F.S., J.F., P.M., and B.G. searched articles published in English until February 2025 and selected them on the basis of inclusion and exclusion criteria. In order to synopsize the scoping review process, a flow diagram was created. Reports of the scoping review were carried out on the basis of a guidance for authors when choosing a scoping review approach [22,23], as shown in Figure 1. This review followed the PRISMA guidelines. We registered our scoping review on OSF Registries with the following registration link: https://doi.org/10.17605/OSF.IO/NZXYQ (accessed on 20 February 2025).

## 3. Effects of Vitamin D and Its Analogues

### 3.1. Vitamin D and Its Analogues

VitD belongs to the group of fat-soluble vitamins. Its blood levels are the result of a combination between cutaneous synthesis through UV irradiation and intake with food or VitD supplements [11]. Its main circulating metabolite, 25-hydroxyvitamin D [25(OH)D], derives from the hydroxylation on position 25 of VitD in liver via 25-hydroxylase encoded by the gene *CYP2R1*. Then, 25(OH)D is bound to the VitD-binding protein and transported to the target tissues, mainly in kidneys, where a further hydroxylation on position 1, via 1α-hydroxylase coded by the *CYP27B1* gene, occurs [24,25]. VitD can act both as a vitamin and a hormone, and its effects are mediated by the VitD receptor (VDR), an intracellular receptor member of the family of nuclear hormone receptors. VDR is formed by a DNA-binding domain that binds to a VitD response element (VDRE) by a C-terminal ligand-binding domain and by a third region that connects these two domains [26]. VitD interaction with VDR is responsible for its genomic effects. It can also exert non-genomic effects on different cell types via a membrane receptor. One of these effects is the increase in calcium and phosphate intestinal re-uptake, a process named transcalcification [24]. VitD also regulates the activity of chloride channel, stimulates protein kinase C and phospholipase C in different cellular types, such as osteoblasts, hepatocytes, and intestinal cells, and promotes the renal reabsorption of phosphate and the passage into the blood of calcium from the bones [27]. In the human body, blood levels of 25(OH)D are considered as a dependable estimation of the VitD status. Commonly, VitD levels between 50 and 75 nmol/L are considered as VitD insufficiency, while levels < 50 nmol/L are considered as VitD deficiency (VDD) [28]. VitD insufficiency status is a worldwide diffuse condition [29]; in particular, 20% of Afghanistan, Pakistan, Tunisia, and India people have lower levels (below 30 nmol/L). In this regard, epidemiological studies have suggested an increased risk of different types of cancers in patients with VDD [30,31] and, recently, an association between VitD levels and prognosis for cancer patients [32].

As above indicated, the main function of VitD is to maintain the proper levels of calcium and phosphorus in serum. Besides its major role of maintaining calcium–phosphate balance, it regulates most events, typical of cancer, being able to inhibit cellular proliferation, neoangiogenesis, and metastasis, to induce cellular differentiation and apoptosis, and to control the immune system [33].

It was observed that high doses of VitD provided anticancer activity and can cause hypercalcemia as a side effect. For this reason, various synthetic VDAs with a low calcemic effect versus a powerful antiproliferative, differentiating, and/or immune-modulatory role have been developed [34]. These drugs are created with structural modifications of the basic structure of VitD so that they can act more efficiently and more selectively, with reduced side effects.

However, conflicting data are present in the literature on the use of VDAs. In fact, some studies have described an elevated risk of complications (hypercalcemia, hypercalciuria, and hyperphosphatemia) when VDAs are used, compared to cholecalciferol and ergocalciferol, so that the use of VDAs should be cautiously evaluated and controlled, particularly in patients at a high risk of these complications [35]. On the contrary, other studies [36] indicated that some of the about 3000 synthetic VDAs produced by different pharmaceutical companies and research groups may interfere with the proliferation of the cancer cells in different cell lines, owing to their binding affinity to VDR. However, even if in human pancreatic tumors the VDA calcipotriol strikingly reduced the markers of inflammation and fibrosis [37], the positive activity of VDR ligands is still primarily referred to in vitro assays.

### 3.2. In Vitro Effects of Vitamin D and Its Analogues

As to the in vitro studies (Table 1), it was demonstrated that 1,25(OH)2D3 and certain VDAs displayed cytotoxic and pro-apoptotic actions upon DLBCL cells.

With its high invasiveness, quick progression, and ease of spread, DLBCL is the most prevalent clinical subtype of aggressive non-Hodgkin’s lymphomas [37]. Based on statistical estimations, more than 40% of aggressive lymphomas are caused by DLBCL. According to reports, there are over 150,000 new DLBCL cases worldwide each year [43]. Sadly, relapse or refractory disease will occur in around 40% of DLBCL patients who are not responsive to conventional chemoimmunotherapy [44].

In vitro, these compounds caused the death of approximately 40% of DOHH2 cells, a cell line representative of DLBCL, after 24 h stimulation. Furthermore, 1,25(OH)2D3 and VDAs increased the expression of VDR protein in DOHH2 cells, which suggested a pronounced biological activity of these compounds upon malignant B-cell disease. Moreover, this study revealed that VitD3 and analogues can enhance the anti-proliferative efficacy of various chemotherapeutics, such as clomipramine [35].

In another study, Han et al. [38] demonstrated that, in the DLBCL Pfeiffer cell line, the subtype of non-Hodgkin lymphoma, calcitriol with the macrolide Rapamycin (RAPA) blocked proliferation, induced an increase in cells in G1 phase and amplified the cell-cycle arrest. It was also demonstrated that RAPA and calcitriol suppressed the expression of VDR and of 25-hydroxyvitamin d-24-hydroxylase (CYP24A1), the enzyme that causes 1,25(OH)2D3 degradation.

Several effects of VitD have been identified on immune cells such as T-, B-, NK-, monocytes and dendritic cells (DCs), as well as their involvement in tumors and autoimmune diseases [45,46,47,48].

Neumann et al. [39] showed VitD3 serum levels associated with maximum NK=cell-mediated antibody-dependent cellular cytotoxicity (ADCC). In fact, NK-cell-mediated ADCC is the major mechanism of action of both rituximab and obinutuzumab, whose efficacy improved significantly after VitD3 supplementation in VitD3 deficient and insufficient patients. In particular, obinutuzumab showed a stronger ADCC activity than rituximab. Therefore, the effect of VitD3 on NK-cell-mediated ADCC is crucial and can improve the clinical outcome of immunotherapies based on these antibodies.

In line with these results, Bold et al. [40] showed immune modulatory effects of calcitriol on stimulated NK-cells in vitro. More in detail, NK-cells isolated from healthy volunteers, co-incubated with B-cell lymphoma DAUDI and U2932, stimulated with Interleukine-2, and treated with calcitriol by long-term stimulation presented an increased ADCC against these tumor cells.

Similar results were found in a different lymphoproliferative disorder such as HL. This disease accounts for approximately 30% of all malignant lymphomas in Europe and the United States [49]. Gharbaran et al. [41] demonstrated that HL-cell lines and primary Hodgkin and Reed/Sternberg cell lines showed low levels of VDR expression. In addition, VitD3 and VDAs (calcipotriol and EB1089) reduced the growth of HL-cell lines, associated with an increased accumulation of VDR, which induced and controlled the expression of genes.

However, the active forms of VitD3 and its analogues are not efficacious in all cancer cells, even if they show high levels of VDR expression [42]. The response to calcitriol and to the analog tacalcitol (PRI-2191) in leukemia and lymphoma cell lines could be influenced by the VDR polymorphism, especially Fok1 polymorphism and “bat” haplotype.

### 3.3. In Vivo Effects of Vitamin D

Several in vivo studies focused their attention mainly on DLBCL (Table 2).

The influence of VDD on the therapeutic outcomes in elderly patients with DLBCL was investigated [50]. Patients with VitD levels ≤ 8 ng/mL had an event-free survival (EFS) of 59% and an overall survival (OS) of 70%, while patients with VitD levels > 8 ng/mL showed an EFS and OS of 79% and 82%, respectively.

In another study [51], plasma total 25(OH)D and bioavailable 25(OH)D levels at diagnosis in 332 newly diagnosed DLBCL patients were examined; the results were correlated with clinical characteristics, prognosis, and response to treatment. VDD (25-(OH)D < 30 ng/mL) was found in 92.8% of patients. Higher values of plasma bioavailable 25(OH)D were correlated with better progression-free survival (PFS) and OS, while higher levels of plasma total 25(OH)D significantly influenced PFS but not OS.

A lower PFS and OS were reported by Wang et al. [52], who investigated the prognostic value of VDD (25-(OH)D < 52.5 nmol/L) in 208 newly diagnosed DLBCL patients. It was shown that 25-(OH)D deficiency was an independent prognostic predictor for worse PFS and OS.

Nath et al. [53] evaluated the association between pre-therapy VitD levels and PFS, OS, and CAR-T-related toxicity and response in 111 relapsed/refractory DLBCL patients. In this study, 73 patients presented VDD (≤30 ng/mL), and they had worst 100-day complete response and 2-year OS.

Drake et al. [54] evaluated if circulating 25(OH)D levels influenced EFS and OS in a prospective cohort of 983 newly diagnosed patients with NHL. They reported lower EFS and OS in DLBCL patients with VDD, while they did not observe association between EFS and other NHL subtypes. Moreover, among patients with DLBCL, higher VitD levels were correlated with better EFS and OS.

Another type of B lymphoma is follicular lymphoma (FL), an indolent lymphoid tumor generated from germinal center B-cells. Recent developments in treatment for FL have significantly increased patient life. However, FL is still an incurable illness, nonetheless, with some patient groups exhibiting early disease development, histologic transformation, or a significant risk of damage from treatment. Furthermore, response rates and disease-control intervals decline with each new line of treatment for FL, which is a recurrent disease [61].

Two studies observed a worse prognosis in FL patients with VDD. In the first one [55], the impact of pretreatment with 25(OH)D on PFS between two independent cohorts of similarly treated prospective patients with newly diagnosed FL was evaluated. Amongst the patients with VDD < 20 ng/mL, the median follow-up was 5.4 years, while those with VDD < 10 ng/mL had a median follow-up of 6.6 years.

In the second study [56], the association between VDI (25(OH)D < 20 ng/mL) and adverse outcomes in FL patients was assessed. They observed a lower OS and EFS at 12 months in patients with VDI for the full cohort.

Another study by Eicher et al. [57] investigated the effect of VitD serum levels on PFS and OS in patients with different types of lymphomas undergoing high-dose chemotherapy (HDCT)/autologous stem cell transplantation (ASCT). It was shown a better PFS and OS in patients with VitD levels > 52 nmol/L.

Xu et al. [58] studied the prognostic value of VDD in patients with mantle cell lymphoma (MCL), another subtype of B-cell NHL. MCL is a rare type of B-cell neoplasm characterized by the growth of mature B-cells, typically expressing CD5. It predominantly affects older males, with a median age of around 65 years. Although it is generally classified as aggressive, it exhibits a wide range of clinical behaviors [62].

Qin et al. [59] studied the influence of serum 25-(OH)D deficiency on PFS and OS in 77 newly diagnosed HL patients. They observed that patients with VDD had lower PFS and OS compared with patients with normal VitD levels.

Moreover, Borchmann et al. [60] measured VitD levels before treatment in 351 prospectively treated patients with HL, correlating these results with their clinical outcomes. They found VDD in 50% of patients, being lower levels more common in relapsed/refractory patients. Moreover, patients with VDD had consistently impaired PFS.

### 3.4. Diet and Vitamin D

A limited number of studies have addressed associations between diet and risk for lymphoma, as well as the impact of VitD supplementation in this context.

Overall, in light of the scarcity of clinical evidence and contrasting results, “promotion of healthy lifestyles and empowering of lymphoma survivors should be implemented”, as suggested by Fondazione Italiana Linfomi researchers [63]. Indeed, in their systematic review, they concluded that the areas of development of clinical research are numerous, and it is wanted to implement plans encouraging and instructing patients on healthier lifestyles, such as consuming a healthy diet. Intriguingly, to date, VitD and other nutraceutical compounds might positively impact lymphoma prognosis even if safety issues should be carefully considered especially for the concomitant use of other dietary supplements and lymphoma-directed therapies [64].

## 4. Discussion and Conclusions

Lymphoma is the most common hematological malignancy in developed countries, whose evolution is induced by alterations in the hematopoietic cells. At present, although many strategies have been proposed for the treatment of these pathologies, myeloid and lymphoid cancer therapies are still not very successful. Therefore, researchers have focused their attention on new strategies and therapy options to increase the efficiency and tolerance of standard treatments, such as chemotherapy, radiotherapy, and immunotherapy. Amongst these strategies, calcitriol has shown an important role in the pathogenesis, progression, and therapy of hematological cancers. We reviewed both in vitro and in vivo studies that evaluated the influence of VitD and its analogs on prognosis and treatment efficacy in B lymphomas.

**Figure 2:**
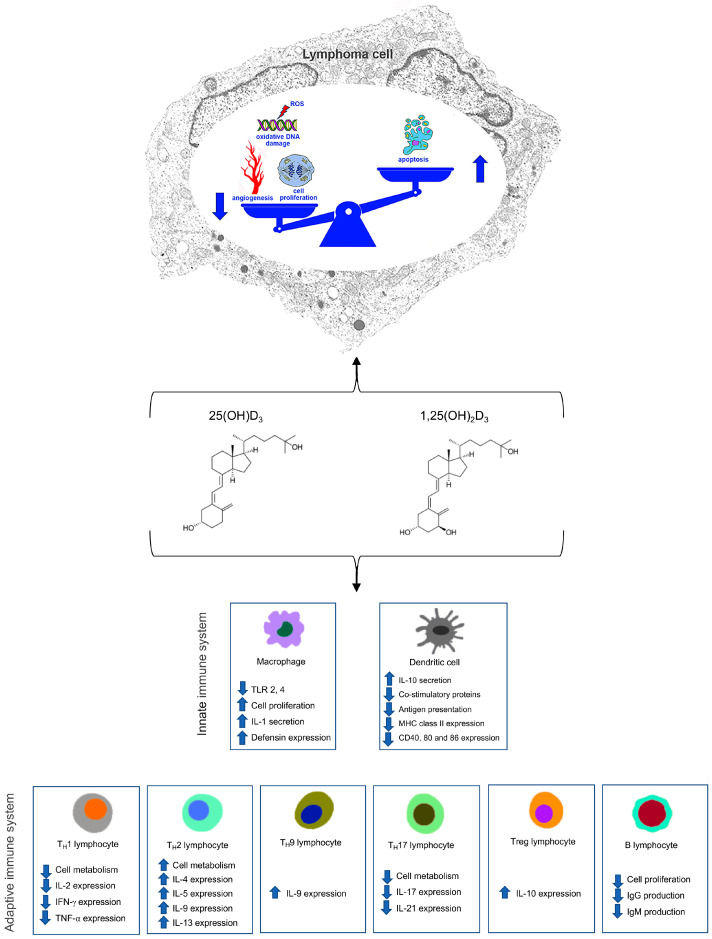
Schematic representation of main actions of Vitamin D on lymphoma cell and on cells of the innate and adaptive immune system. 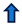 Increased; 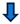 Decreased.

The in vitro studies revealed cytotoxic and pro-apoptotic effects of calcitriol and VDAs on NHL-cell lines, blocking proliferation and inducing the G1 phase or cycle cell arrest [38]. Besides controlling cancer cells’ viability directly, calcitriol and VDAs act also indirectly on cells of the innate and adaptive immune system, including macrophages as effector cells [65] with negative effects on tumor cells (Figure 2).

The treatment with VitD and its analogues enhances the effect of different drugs such as antibiotics, chemotherapeutics, and biological drugs. In fact, these compounds, in co-administration with rapamycin, amplified cell-cycle arrest in Pfeiffer cell line [38]. The concomitant treatment with Obinutuzumab and calcitriol on NK-cells isolated from healthy volunteers stimulated an increased ADCC of these cells against B lymphoma cells [39].

Thus, the way by which lymphomas react to treatment may also be influenced by VitD. As reported above, it has been demonstrated that VitD actually increases the cellular cytotoxicity induced by rituximab [50]. In fact, deficiency in 25-hydroxyvitamin-D (25-OHD) has been found to be a poor prognostic indicator in patients receiving chemotherapy for follicular lymphoma and newly diagnosed DLBCL [54,55]. Moreover, better results in DLBCL have been linked to supplementation-achieved stabilization of VitD levels [13]. A phase III clinical trial (NCT03078855) is currently investigating the combination of VitD supplements and rituximab in non-Hodgkin lymphoma.

Finally, the management of relapsed/refractory large B-cell lymphoma has advanced dramatically as a result of immunotherapy combined with chimeric antigen receptor T-cell therapy (CAR-T), and three anti-CD19-CAR-T medicines are currently licensed for this indication [66]. According to a study, CAR-T recipients who are VitD insufficient had worse clinical outcomes [53].

Ultimately, consolidation for patients with recurrent lymphomas involves the use of autologous stem cell transplantation (ASCT) along with high-dose chemotherapy (HDCT). In a study, the impact of serum levels of VitD on progression-free survival (PFS) and overall survival (OS) was examined in patients with lymphomas undergoing ASCT. Higher VitD levels (>52 nmol/L) were linked to improved OS and PFS. Based on these findings, it appears that lower serum VitD levels are linked to worse outcomes for patients with lymphoma undergoing HDCT/ASCT. Prior to ASCT, VitD level optimization might be necessary [57].

Finally, another crucial anti-cancer activity of these substances is the interaction with VDR, a receptor generally largely expressed by cancer cells. In line with this knowledge, it has been shown that in the presence of a decreased expression of VDR, the treatment of high-grade B-cell lymphoma lines with higher concentrations of calcitriol induced a slightly appearance of this receptor [67].

Regarding the in vivo studies, they confirmed the positive influence of VitD on lymphomas prognosis and therapy efficacy [50]. In fact, patients with VDD always presented a worse PFS and OS when compared to patient with VitD levels within range.

These results confirm what several works already reported regarding VitD and its role in other types of solid tumors. In fact, a significant inverse association between VitD levels and breast, prostate, and colon cancer prognosis has already been found [68].

As to the possible explanation of the better outcomes in patients with high VitD levels, VitD and its analogues might modulate the tumor microenvironment reducing cancer prevention and progression [69,70]. In fact, cancer patients with higher VitD levels can have better general health conditions that might lead to improved overall survival [71]. By contrast, even if cancer tumors lead to extra-renal production of 1,25(OH)2D3 to fight the tumor [72,73], it has been found that 1,25D analogues are not useful in treating cancer [74].

So far, in the last years, it has been particularly focused on the role of additional factors as hypercalcemia in patients with lymphoma. It has been suggested that the major mechanism by which NHL patients develop hypercalcemia is not mediated by calcitriol or parathyroid-related protein. As a matter of fact, hypercalcemia is most prevalent in patients with diffuse large B-cell lymphoma of the nongerminal cell subtype. Overall, multiple evidence indicates that patients with calcitriol-mediated hypercalcemia showed a trend toward worse outcomes, suggesting that calcitriol might be a marker of high-grade lymphoma or a surrogate for more advanced disease [75,76,77,78]. Interestingly, in malignant lymphoma causing hypercalcemia, Ogawa and colleagues encountered a case of hypercalcemia induced by the overproduction of 1,25(OH)2VitD in DLBCL. They showed that immunostaining with the anti-CYP27B1 antibody was useful for identifying the ectopic expression of 1α-hydroxylase in lesions and facilitated the selection of corticosteroids as the treatment to control hypercalcemia in malignancy [79]. Indeed, evidence to support the excessive synthesis of 1,25(OH)2VitD by the ectopic expression of 25-hydroxyvitamin D3-1α-hydroxylase (1α-hydroxylase) in malignant lymphomas is limited [80,81]; however, the current research focused, in the management of the above conditions, on other pharmacological approaches such as hydration, elcatonin, bisphosphonates, and denosumab and, in some patients, corticosteroids and cinacalcet. Specifically, it is important to keep in mind that corticosteroids are effective not only in the treatment of lymphoma but also for 1,25(OH)2VitD-mediated hypercalcemia [79].

Indeed, VDD is associated with many pathological conditions, such as cancer, diabetes, autoimmune, cardiovascular, and infectious diseases [82].

Because VitD affects the immune system and the ability to fight off infections, it may be especially helpful for people with lymphoproliferative diseases. Even nonfatal infections can interfere with chemotherapy regimens and have crippling long-term effects [83], and patients with lymphoproliferative diseases are still at a high risk of serious and life-threatening infections [84,85] despite the widespread adoption of infection prevention practices.

The finding that VitD deficiency is linked to an increased risk of infections is consistent with previous research showing that VitD supplementation may provide protection against infection [86]. Previous studies showed that nutritional deficiencies, particularly VitD deficiency, may contribute to the risk of infection by causing complex multisystem effects, including immunological defects. VitD actually affects how B-cells, T-cells, innate immune cells, and epithelial cells form, survive, and function [87]. Additionally, VitD forms endogenous AMPs called defensins through cytokine pathways, which kill invasion organisms inside cells [88].

Moreover, human B-cell development and function are impacted by VitD receptor activation. Through its nuclear receptors, 1,25-dihydroxyvitamin D3 influences B-cell migration and homing, immunoglobulin class switching to IgA at the expense of IgE, and B-cell differentiation. The effects of vitamin metabolites on B-cell survival factors, such as the control of BAFF and APRIL, the stimulation of TGF-β, or the inhibition of NF-κB, are well understood. Naïve B-cells differentiate into IgA+ plasmablasts when exposed to calcitriol [89].

Since CLL patients are said to have a decrease in antibody production [90,91], and because antibody therapy may benefit them [92], this factor is significant. IgG and IgA class-switching through abnormal CD40–CD40 ligand relations of and reduction in CD40 ligand; the suppression of CD95+ plasma cells in the bone marrow through interaction with CD95 ligand on CD5-B-cells; disproportionate inhibition by T-cells; iatrogenic myelosuppressive treatment; and the malfunctioning generation of polyclonal immunoglobulins and anomalous activity of non-neoplastic CD5-B-cells are among the causes of poor immunoglobulin concentrations [93,94].

Cesur et al. showed that, depending on the length, VitD use considerably raised serum IgG levels in comparison to individuals who did not take it and that the study stayed above the baseline over the long run [95]. It has been found that the 25(OH)VitD levels during VitD use have a positive and substantial connection with the most recent measurement of blood IgG. The study found that blood IgG levels remained above the initial level for a considerable amount of time and rose significantly based on the length between those who utilized VitD and those who did not.

As VitD administration continued, a positive and substantial correlation between the latest measured immunoglobulin G and 25(OH)D levels was seen [95].

The information presented above is especially pertinent to some patient subgroups, such as those infected with SARS-COVID-19. Relapsed immunocompromised COVID-19 patients who have had CD20 depleting therapy may benefit from combined therapy using selective intravenous immunoglobulin and antiviral medications, according to a recent study. The virus load dropped without reoccurring after the patient underwent intravenous immunoglobulin treatment [96]. VitD supplementation may enhance immunoglobulins’ protective function and enhance the prognosis of these individuals, as, while VitD supplementation may lessen the severity of a patient’s illness, low VitD levels are linked to increased infection rates, more severe disease, and higher fatality rates among COVID-19 patients [97].

In conclusion, the data in our possession suggest that higher levels of circulating VitD is associated with improved OS, reduced cancer-specific mortality, and better disease-free survival for various cancer types, with particular regard to lymphomas. Furthermore, VitD and analogs showed also positive effects in in vitro studies, while only VitD was able to improve clinical parameters. It is also the case that more studies are necessary to better understand the clinical benefits of VitD supplementation in lymphoma patients and, additionally, to develop model animals and clinical trials on the effects of VDAs in these diseases.

Given the aforementioned, we think that all lymphoma patients should be evaluated and given supplements, even though the exact role of VitD in these subjects is still unclear, particularly with regard to the best dosage to use. Regarding dosing, several different dosages have been suggested for various lymphoproliferative disorders. The authors of a study supplemented patients with VitD according to their age and 25-hydroxyvitamin D levels. Every three months, levels of the goal 25-hydroxyvitamin D level, which was set at ≥30 ng/mL [98], were measured. We believe it is fair to recommend 1000 IU of VitD per day for patients, with greater dosages required to address severe deficiencies. However, there are a lot of real-world issues to deal with, such as VitD poisoning.

It is very uncommon to become intoxicated from regularly prescribed dosages. The majority of intoxication instances are caused by self-initiated supplementation, formulation problems, or unintentional overdoses associated with prescription regimens. However, serious side effects are possible, such as renal failure necessitating hemodialysis [99]. Treatment options for cases of hypercalcemia brought on by excessive exogenous VitD3 supplementation have generally involved daily dosages above 50,000 IU for at least two to three months, along with intravenous fluids, loop diuretics, calcitonin, and glucocorticoids [100].

Lastly, a variety of factors, including age, gender, and ethnicity, must be taken into account. A study looked at VitD levels that were stratified by several factors at once. This approach determined which populations were more susceptible to negative health effects as a result of low VitD levels. For instance, non-Hispanic Black females of reproductive age had significant levels of deficit [101].

Moreover, it appears noteworthy to underline the role of a healthy diet in this context. Actually, limited evidence suggests that low-fat, plant-based diets may reduce the risk of this disease [102,103]. Data from the European Prospective Investigation into Cancer and Nutrition study revealed for the first time that adherence to a Mediterranean diet was modestly associated with a reduced risk of overall lymphoma [103]. However, it is not yet known whether other dietary factors, nutraceuticals, and/or dietary supplements can influence the course of lymphomas as studies aiming to evaluate the impact of the Mediterranean diet and VitD levels on late toxicities and secondary cancers were lacking. However, this aspect should further stimulate the scientific community to pay ever more attention to the promotion of lifestyles aimed at the prevention of late toxicities related to anti-cancer treatments [104].

However, due to its limited solubility in gastrointestinal tract aqueous fluids, VitD is a non-polar lipid with low bioavailability. Better absorption may be made possible by a process called micellization, which distributes fatty compounds into aqueous micellar spheres. Recent studies have demonstrated that VitD3 nanoemulsion formulations based on nanotechnology outperform traditional coarse emulsions in terms of homogeneity and bioavailability. These procedures might enhance biological effects of VitD in hematological patients [105,106].

On the basis of these observations, we trust that a multifaceted approach including a plant-based diet, adequate levels for physical activity [107], and/or eventually supplemented by VitD, could represent a valuable strategy to challenge lymphomas and, more in general, non-communicable diseases and their comorbidities.

## Figures and Tables

**Table 1:** Summary of the in vitro studies.

Study	Cell Line	Results	Reference Number
Kozielewicz et al., 2016	DOHH2	VitD and VDAs had cytotoxic and pro-apoptotic actions, increased VDR expression, and the anti-proliferative efficacy of clomipramine.	[35]
Han et al., 2019	DLBCL Pfeiffer	Calcitriol blocked proliferation and induced G1 phase, and this effect was augmented with the co-administration of RAPA; calcitriol increased autophagy by stopping the PI3K/AKT/mTOR pathway.	[38]
Neumann et al., 2018	DAUDI (CD20^+^ Burkitt’s lymphoma cells)	DAUDI cells cultured with NK-cells obtained from healthy donors and ADCC activity were determined by lactate dehydrogenase release assay. NK-cells killed lymphoma cells in a concentration and E:T ratio-dependent manner with obinutuzumab displaying a stronger ADCC activity than rituximab.	[39]
Bold et al., 2022	DAUDI, U2932	Increased ADCC against tumor cells in cells stimulated with IL-2 and treated with highest concentration of calcitriol.	[40]
Gharbaran et al., 2019	HL, HRS (Hodgkin and Reed-Sternberg cells)	Low VDR expression; reduction in HL-cell line growth after 72 h of treatment with VitD and VDAs at 10 μM concentration; this reduction was associated with an increased VDR nuclear accumulation.	[41]
Gleba et al., 2022	KG-1, K562, HL-60, MV-4-11, Thp-1 Jurkat, DAUDI, Raji	MV-4-11, Thp-1, and HL-60 cell lines sensitive to calcitriol and tacalcitol inhibition on proliferation showed morphological changes.	[42]

Legend: VitD: Vitamin D; VDAs: Vitamin D analogues; RAPA: rapamycin; DLBCL: diffuse large B-cell lymphoma; ADCC: antibody-dependent cellular cytotoxicity; VDR: Vitamin D receptor; HL: Hodgkin’s lymphoma.

**Table 2:** Summary of the in vivo studies.

Study	Number of Patients	Type of Lymphoma	Results	Reference Number
Bittenbring et al., 2014	359	DLBCL	Worst EFS and OS in patients with VDD.	[50]
Chen et al., 2021	332	DLBCL	Worst PFS and better response to treatment in patients with higher VitD blood levels.	[51]
Wang et al., 2020	208	DLBCL	Worst PFS and OS in patients with VDD.	[52]
Nath et al., 2022	111	DLBCL	Worst response to treatment and 2-year OS. In patients with VDD, no correlation between pre-treatment VDD and CAR-T-related toxicity.	[53]
Drake et al., 2010	983	DLBCL	Worst EFS and OS in patients with VDD; no association between EFS and VDD in other NHL subtypes.	[54]
Kelly et al., 2015	1979	FL	Worst PFS in patients with VDD.	[55]
Tracy et al., 2017	642	FL	Worst OS and EFS at 12 months in patients with VDI.	[56]
Eicher et al., 2020	183	Various types of lymphomas	Better PFS and OS in patients with VitD levels > 52 nmol/L.	[57]
Xu et al., 2020	70	MCL	Worst PFS and OS in patients with VDD.	[58]
Qin et al., 2021	77	HL	Worst PFS and OS in patients VDD.	[59]
Borchmann et al., 2019	351	HL	VitD low levels more common in R/R patients; worst PFS in patients with VDD.	[60]

Legend: EFS: event-free survival; PFS: progression-free survival; OS: overall survival; VDD: vitamin-D deficiency; VDI: Vitamin D insufficiency; NHL: non-Hodgkin lymphoma; R/R: relapsed/refractory; DLBCL: diffuse large B-cell lymphoma; FL: follicular lymphoma; MCL: mantle cell lymphoma; HL: Hodgkin’s lymphoma.
